# Mechanical Fault Diagnosis of High Voltage Circuit Breakers Based on Variational Mode Decomposition and Multi-Layer Classifier

**DOI:** 10.3390/s16111887

**Published:** 2016-11-10

**Authors:** Nantian Huang, Huaijin Chen, Guowei Cai, Lihua Fang, Yuqiang Wang

**Affiliations:** 1 School of Electrical Engineering, Northeast Electric Power University, Jilin 132012, China; chjin1990@126.com (H.C.); caigw@mail.nedu.edu.cn (G.C.); zang_fang0412@163.com (L.F.); 2 State Grid Jibei Electric Power Co., Ltd. Maintenance Branch, Beijing 102488, China; wangyq181@163.com

**Keywords:** mechanical fault diagnosis, high voltage circuit breakers, acceleration sensor, variational mode decomposition, local singular value, one-class support vector machines

## Abstract

Mechanical fault diagnosis of high-voltage circuit breakers (HVCBs) based on vibration signal analysis is one of the most significant issues in improving the reliability and reducing the outage cost for power systems. The limitation of training samples and types of machine faults in HVCBs causes the existing mechanical fault diagnostic methods to recognize new types of machine faults easily without training samples as either a normal condition or a wrong fault type. A new mechanical fault diagnosis method for HVCBs based on variational mode decomposition (VMD) and multi-layer classifier (MLC) is proposed to improve the accuracy of fault diagnosis. First, HVCB vibration signals during operation are measured using an acceleration sensor. Second, a VMD algorithm is used to decompose the vibration signals into several intrinsic mode functions (IMFs). The IMF matrix is divided into submatrices to compute the local singular values (LSV). The maximum singular values of each submatrix are selected as the feature vectors for fault diagnosis. Finally, a MLC composed of two one-class support vector machines (OCSVMs) and a support vector machine (SVM) is constructed to identify the fault type. Two layers of independent OCSVM are adopted to distinguish normal or fault conditions with known or unknown fault types, respectively. On this basis, SVM recognizes the specific fault type. Real diagnostic experiments are conducted with a real SF_6_ HVCB with normal and fault states. Three different faults (i.e., jam fault of the iron core, looseness of the base screw, and poor lubrication of the connecting lever) are simulated in a field experiment on a real HVCB to test the feasibility of the proposed method. Results show that the classification accuracy of the new method is superior to other traditional methods.

## 1. Introduction

As an integral part of the power system, high-voltage circuit breakers (HVCBs) are responsible for the control and protection of the system. HVCB faults will directly harm system reliability, causing significant outage costs. Therefore, the study of fault diagnostic methods for HVCBs is urgent. An inquiry about HVCB faults by the International Council on Large Electric Systems (CIGRE) showed that 39% of minor faults and 44% of major faults are of mechanical origin [1]. Hence, the research on mechanical fault diagnosis of HVCBs has practical significance. Vibration signals generated during the opening/closing operations of HVCBs contain certain important information associated with the mechanical state of breakers. Runde et al. [2] demonstrated through an extensive HVCB diagnostic test that vibration analysis is a suitable and reliable noninvasive diagnostic method for HVCBs. Analysis of HVCB vibration signals collected by acceleration sensor has been widely used in the state detection and fault diagnosis of HVCBs [3,4,5,6,7,8,9].

HVCB vibration signals have a strong transient and wide frequency distribution. The signal acquisition equipment must have a high sampling rate. An acceleration sensor has high accuracy, wide frequency and amplitude responses, small size, and is easy to install; thus, it is widely used in vibration data acquisition [3,4,5,6,7,8,9,10,11]. 

Signal processing is conducted after vibration signals are obtained by the acceleration sensor, extracting the signal features for fault diagnosis. The HVCB vibration signals during operation are nonstationary and nonlinear. Traditional signal-processing methods, such as Fourier transform (FT) [12], are unsuitable for vibration signal analysis and processing. Conversely, time-frequency analysis methods, including wavelet packet decomposition (WPD) [3], empirical mode decomposition (EMD) [6,7], and local mean decomposition (LMD) [9], can analyze HVCB vibration signals well. Wavelet analysis can represent the local characteristics of signals both in time and frequency domains; thus, it is widely used in mechanical fault diagnosis [13]. However, wavelet transform is essentially an adjustable windowed FT with the limitation of energy leakage [14]. Wavelet basis function and decomposition scale are difficult to select in practical applications. The EMD proposed by Huang et al. is a completely adaptive signal analysis method, which is suitable for nonstationary signals analysis [15]. However, the EMD algorithm has a problem of mode aliasing. EMD is sensitive to noise and sampling, and its algorithmic nature lacks mathematical theory [16]. The LMD algorithm is similar to EMD; consequently, it also has the above-mentioned disadvantages of the EMD algorithm.

Variational mode decomposition (VMD) is a new adaptive signal-processing method proposed by Dragomiretskiy et al. (2014) [16]. This method introduces an entirely non-recursive VMD model and translates the decomposition problem into a variational one. Each mode and corresponding center frequency are continuously updated by solving the optimal solutions of the variational problem. VMD method has a solid theoretical foundation and good noise robustness. It has been successfully applied to the propagating mode extraction of microwave waveguide [17], the classification of power quality events [18], speech signals, and mechanical fault detection [19,20]. The features of vibration signals can be easily extracted from the IMFs of VMD.

After the feature extraction of fault vibration signals, a classifier should be used for fault type identification. Neural networks (NNs) [3] and SVM [6,7] achieve good classification accuracy in HVCB fault recognition. NNs have better capacities of self-learning and non-linear pattern recognition [21]. However, the determination of various parameters of NNs is difficult, and finding the optimal configuration of NNs is time consuming [22]. SVM algorithm is based on statistical learning theory and structural risk minimizing principle. It is suitable for classification problems with small sample size [23]. Sufficient fault sample data with all fault types are difficult to obtain because HVCB operations are seldom. SVM cannot correctly identify a new fault type because of lack of training samples. Consequently, the sample of an unknown fault type is recognized as a normal sample. In this case, the SVM-based classifier hardly meets the reliability requirements. The occurrence of a new fault is also unpredictable in an HVCB operation. Recent research results show that only a few types of HVCB mechanical fault with training samples can be recognized [3,6,7]. Thus, a new fault type in the mechanical system cannot be identified successfully.

OCSVM [24] is a classifier that can be trained by using only one type of samples. It is widely used in the field of fault diagnosis and detection [25,26,27,28]. The classification boundary of OCSVM is closer to the object samples than that of SVM. Hence, OCSVM has a lower false acceptance rate, i.e., lower possibility of no-object samples misrecognized as object samples. Accordingly, OCSVM has a superior fault detection capability for HVCBs. 

This paper proposes a new method based on VMD and MLC for diagnosing HVCB mechanical faults. An acceleration sensor is used to acquire HVCB vibration data. The vibration data are then decomposed by VMD to obtain the corresponding IMFs. On this basis, local singular value decomposition (LSVD) is utilized to extract the vibration features. The MLC used for fault recognition is constructed by two OCSVMs and an SVM. The first OCSVM (OCSVM1) trained by normal samples determines whether a test sample is in the fault state. The second OCSVM (OCSVM2) trained by all available fault samples identifies whether the type of the fault samples is new. SVM is adopted to identify the known fault type. Comparative experiments are designed with the measured fault data of real HVCBs to validate the new method.

## 2. Vibration Data Acquisition and Fault Diagnosis Process

### 2.1. Acceleration Sensor

Acceleration is a physical quantity that characterizes an object’s movement. Vibration essence is the reciprocating movement of an object. Thus, vibration data can be obtained by measuring the acceleration with an acceleration sensor. Integrated electronics piezo electric (IEPE) acceleration sensor is widely used and can obtain HVCB vibration signals well. It also has the following advantages: small size, light weight, low noise, and anti-interference capability. This paper adopts a CA-YD-182A piezoelectric acceleration sensor to measure HVCB vibration data. The main technical indicators of the CA-YD-182A include ±250 g (g = 9.8 m/s^2^) measuring range, 20 mV/g sensitivity, 40 kHz natural frequency, 10 kHz frequency response, a maximum output voltage of 6 V, and a weight of 9 g.

### 2.2. Data Acquisition System

**Figure 1:**
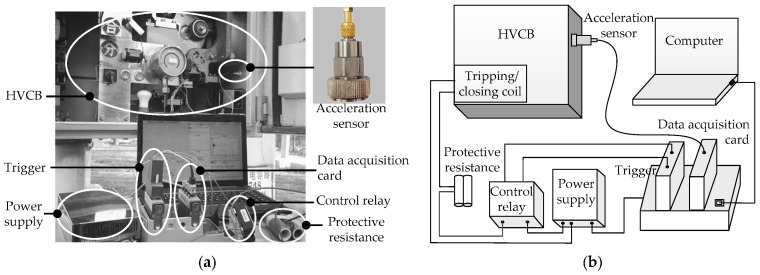
(**a**) The vibration signal acquisition system of HVCBs; (**b**) The block diagram of the acquisition system.

In this paper, the CA-YD-182A acceleration sensor and an NI 9234 data acquisition card are applied to build the vibration signal acquisition system for HVCBs. The measuring object is the LW9-72.5 series, which is an outdoor high-voltage SF_6_ circuit breaker. The acquisition system of HVCB vibration signals and its block diagram are shown in Figure 1. The acceleration sensor is used to measure the vibrational state of HVCBs and produce the corresponding voltage signals. The voltage signals are digitized by using the NI 9234. When the circuit breaker receives an opening command, the system starts sampling. The sampling rate is 25.6 kS/s, and the sampling period is 150 ms.

In an actual measurement, the installation location and the method of the acceleration sensor affect the performance of the acquisition system. The principle for selecting measurement position is that the sensor does not affect the normal operation of the measured object, and the position is close to the object or the most concerned point of the object. In this paper, the sensor is installed on the mechanism box near the operating mechanism. Acceleration sensor installation methods mainly include handheld magnetic adsorption, glue bonding, and screw fixation. An adhesive mounting is selected according to the actual demand of the diagnosis of HVCB mechanical fault.

### 2.3. Fault Diagnosis Process

**Figure 2:**
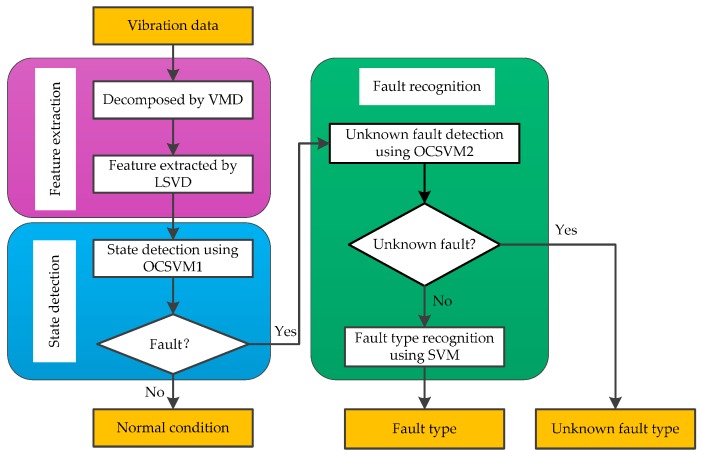
Fault diagnosis process of the proposed method.

The new method proposed in this paper consists of three parts: feature extraction, state detection, and fault recognition. In feature extraction, the features of vibration signals are extracted by using VMD and LSVD methods. In state detection, the normal or fault state of the HVCB is determined by OCSVM1. In fault recognition, the fault type is recognized using OCSVM2 and SVM. The fault diagnosis process is shown in Figure 2, in which OCSVM1 is trained by the normal samples, and OCSVM2 is trained by all available fault samples (fault samples with known types). For a test sample, the new method recognizes a fault as a normal condition, known with a specific fault type, or unknown without specific fault type. 

## 3. VMD

### 3.1. VMD Theory

VMD process is the solving of a variational problem. Therefore, this algorithm can be divided into the construction and solution of the variational problem. VMD involves three key concepts: classic Wiener filtering, Hilbert transform, and frequency mixing.

- Construction of the variational problem
  The VMD turns an input signal *h* into *K* modes. Each mode *m_k_* is mostly compact around a center frequency *ω_k_*. The variational problem can be described as seeking the *K* modes to make the sum of all bandwidths of the modes minimum. The constraint condition is that the sum of each mode is equals to the input signal *h*. The detailed construction scheme is as follows: (1) The associated analytic signal of each mode *m_k_* is computed by the Hilbert transform to obtain the unilateral frequency spectrum; (2) The frequency spectrum of each mode is tuned to the respective estimated center frequency by mixing with the exponential $e^{-j{\omega_{k}}^{t}}$; (3) The bandwidth is estimated through the squared *L*^2^-norm of the gradient of the demodulated signal. The constrained variational problem is written as:
  (1) $$\left\{ \begin{array}{l} \underset{\left\{m_{k}\right\},\left\{\omega_{k}\right\}}{\min}\left\{\sum_{k=1}^{K}{\left\|\partial_{t}\left[\left(\delta \left(t\right)+\frac{j}{\pi t}\right)*m_{k}\left(t\right)\right]e^{-j\omega_{k}t}\right\|}_{2}^{2}\right\}\\ s.t.\;\sum_{k=1}^{K}m_{k}=h \end{array}\right.,$$
  where $\left\{m_{k}\}=\{m_{1},m_{2},\cdots ,m_{K}\right\}$ is the set of all modes, $\left\{\omega_{k}\}=\{\omega_{1},\omega_{2},\cdots ,\omega_{K}\right\}$ are the corresponding center frequencies, δ(t) is the Dirac function, and * denotes the convolution.
- Solution of the variational problem
  A constrained variational problem can become unconstrained by introducing a Lagrange multiplier α and a quadratic penalty factor *η*. The Lagrange multiplier enforces constraints strictly; and the quadratic penalty factor guarantees the reconstruction fidelity of the signal with Gaussian noise. The augmented Lagrange expression is as follows [29]:
  (2) $$L\left(\left\{m_{k}\right\},\left\{\omega_{k}\right\},\alpha \right)=\eta \sum_{k=1}^{K}{\left\|\partial_{t}\left[\left(\delta \left(t\right)+\frac{j}{\pi t}\right)*m_{k}\left(t\right)\right]e^{-j\omega_{k}t}\right\|}_{2}^{2}+{\left\|h\left(t\right)-\sum_{k=1}^{K}m_{k}\left(t\right)\right\|}_{2}^{2}+\langle \alpha \left(t\right),h\left(t\right)-\sum_{k=1}^{K}m_{k}\left(t\right)\rangle ,$$
  The alternating direction method of multipliers (ADMM) solves the saddle point of the augmented Lagrange. $m_{k}^{n+1},$ $\omega_{k}^{n+1},$ and $\alpha^{n+1}$ are alternately updated using the ADMM approach. The updates of $m_{k}^{n+1},$ $\omega_{k}^{n+1},$ and $\alpha^{n+1}$ are as follows (see Appendix A for the detailed solution process):
  (3) $${\hat{m}}_{k}^{n+1}\left(\omega \right)=\frac{\hat{h}\left(\omega \right)-\sum_{i\neq k}{\hat{m}}_{i}\left(\omega \right)+\frac{\hat{\alpha }\left(\omega \right)}{2}}{1+2\eta {\left(\omega -\omega_{k}\right)}^{2}},$$
  (4) $$\omega_{k}^{n+1}=\frac{\int_{0}^{\infty }\omega {\left|{\hat{m}}_{k}\left(\omega \right)\right|}^{2}d\omega }{\int_{0}^{\infty }{\left|{\hat{m}}_{k}\left(\omega \right)\right|}^{2}d\omega },$$
  (5) $$\alpha^{n+1}=\alpha^{n}+\tau \;\left(h-\sum_{k=1}^{K}m_{k}^{n+1}\right),$$
  where $\hat{\cdot }$ denotes the FT of ·, and τ is the update parameter of the Lagrange multiplier. The mode $m_{k}^{n+1}$ can be obtained as the real part of the inverse FT of ${\hat{m}}_{k}^{n+1}$. VMD estimates the mode *m_k_* and center frequency *ω_k_* constantly through an iteration. For a given convergence tolerance *e* > 0, the termination condition of this iteration is:
  (6) $$\sum_{k}{\left\|m_{k}^{n+1}-m_{k}^{n}\right\|}_{2}^{2}/{\left\|m_{k}^{n}\right\|}_{2}^{2}<e,$$

### 3.2. Simulated Vibration Signal Analysis Based on VMD

**Figure 3:**
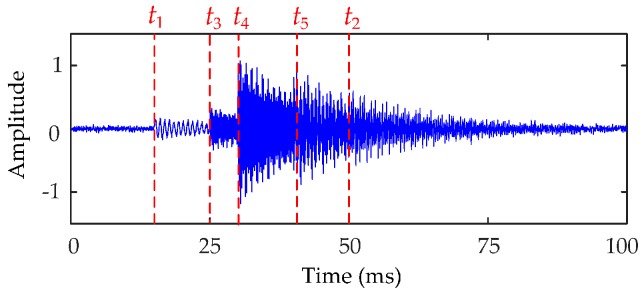
The waveform of the simulated HVCB vibration signal.

The vibration signal of HVCBs consists of a series of vibration events. It can be described by a set of exponentially decaying sinusoidal signals, which is as follows [5]:

(7) $$V\left(t\right)=\sum_{i=1}^{n}A_{i}e^{-\mu_{i}\left(t-t_{i}\right)}\sin\left[2\pi f_{i}\left(t-t_{i}\right)\right]\;\varepsilon \left(t-t_{i}\right),$$

where *n* is the number of vibration events, *ε*(*t*) is the unit step function, *A_i_* is the amplitude of the *i*th vibration event, *μ_i_* is attenuation coefficient, *f_i_* is oscillation frequency, and *t_i_* is the starting time of vibration. The vibration events *V*_1_ to *V*_5_ generated by MATLAB compose the simulated vibration signal for HVCBs. The parameter of each vibration event is shown in Table 1. The waveforms of the simulated vibration signal and each vibration event with a signal-to-noise ratio (SNR) of 20 dB are shown in Figure 3, in which the sampling rate is 25.6 kS/s.

**Figure 4:**
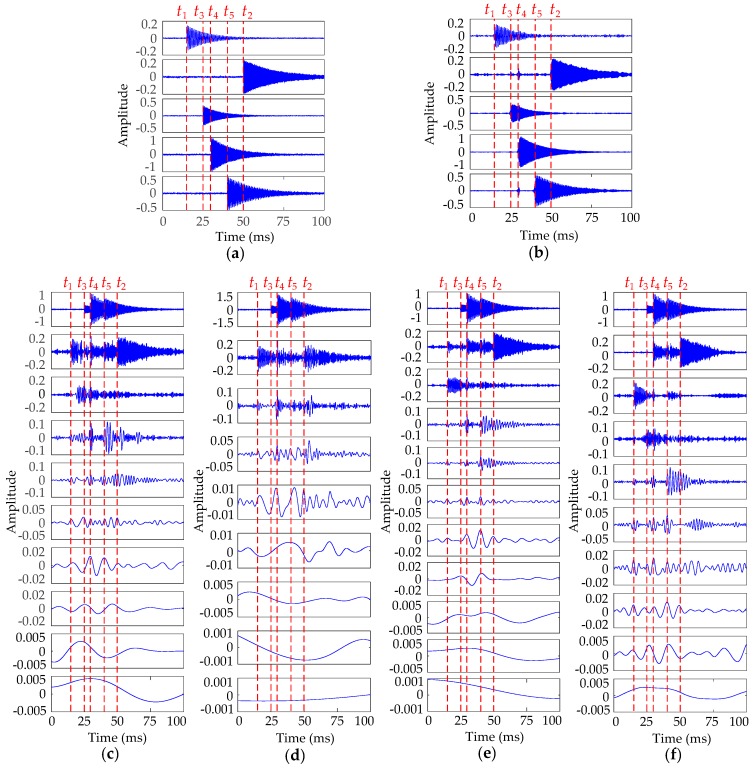
(**a**) Original vibration events; (**b**) IMFs decomposed by VMD method; (**c**) IMFs decomposed by EMD method; (**d**) PFs decomposed by LMD method; (**e**) IMFs decomposed by EEMD method; (**f**) IMFs decomposed by CEEMD method.

EMD has been proven to be a suitable method for the vibration signal processing of HVCBs. We mainly compare the performances of VMD and EMD to decompose this simulated vibration signal (with an SNR of 20 dB). In addition, VMD performance is also compared with a few new and improved EMD-related methods, i.e., LMD [9], ensemble EMD (EEMD) [30], and complete EEMD (CEEMD) [31]. The original vibration events and IMFs decomposed by these five methods are shown in Figure 4.

When the number of vibration events and corresponding parameters of the simulated signal are known, the performance of each signal-processing method can be determined by comparing the correlation degrees of their modes and the original vibration events. Figure 4b shows that the signal is decomposed into five IMFs by VMD, and each IMF is mostly the same as the corresponding vibration event in Figure 4a. That is, the VMD approach can decompose vibration signals thoroughly. Conversely, we obtain approximately 10 IMFs through EMD, LMD, EEMD, and CEEMD approaches. In Figure 4c, the modes decomposed by EMD have a serious mode aliasing problem, especially for the second mode. Although LMD is better than EMD in some aspects, such as the endpoint effect suppression and algorithm speed, it shows almost the same performance as EMD with modal aliasing in this study. Both EEMD and CEEMD can eliminate modal aliasing to a certain extent, but the latter has a better effect. EMD and its derivation algorithms cannot effectively separate the vibration events from the composite vibration signal because of the limitation of its algorithmic nature. Consequently, the characteristics (such as starting time and spectrum) of each mode obtained by EMD and other similar methods are almost irrelevant with the original signal characteristics; thus, these modes fail to reflect the physical significance of each vibration event, i.e., existence of false modes. Therefore, the VMD method is more suitable for the feature extraction of HVCB vibration signals.

### 3.3. Determining the Number of K Modes of VMD

The number of *K* modes should be predefined in VMD method. Each mode component of VMD contains local features of the original signal at a center frequency and different time scales. A great number of *K* modes suggests that VMD has abundant frequency components. The reconstructed signals by *K* modes will be highly similar to the original signal. The measured vibration signals of HVCBs contain a large number of vibration components; thus, the analysis should focus on the main vibration event rather than all vibration components. Therefore, we determine the number of *K* modes by comparing the similarity measure between the reconstructed and original signals.

Distance measure is a common measure of pattern similarity. Normalized distance (ND) is selected to evaluate the similarity between the original and reconstructed signals using different mode numbers. The ND of two discrete signals ***p*** = (*p*_1_,*p*_2_,…*p_n_*) and ***q*** = (*q*_1_,*q*_2_,…*q_n_*) is defined as:

(8) $$d\left(p,q\right)=\frac{\left\|p-q\right\|}{\left\|p\right\|+\left\|q\right\|}=\frac{{\left[\sum_{i=1}^{n}{\left(p_{i}-q_{i}\right)}^{2}\right]}^{1/2}}{{\left(\sum_{i=1}^{n}{p_{i}}^{2}\right)}^{1/2}+{\left(\sum_{i=1}^{n}{q_{i}}^{2}\right)}^{1/2}},$$

**Figure 5:**
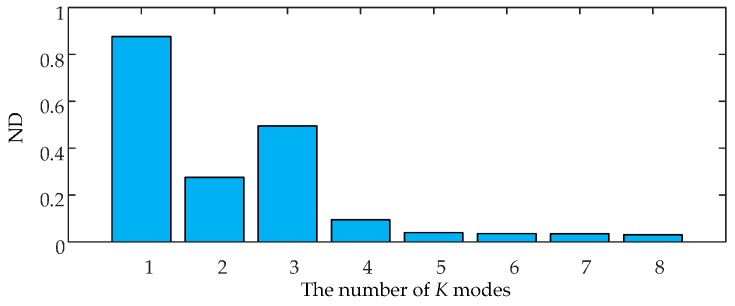
The NDs between the reconstructed signals and the original signals with different *K*.

VMD is used to decompose the simulated vibration signal with different *K* and compute the corresponding reconstructed signals. The NDs between the reconstructed and original signals with different *K* are shown in Figure 5.

Figure 5 shows that the ND almost does not change when *K* becomes greater than 5 and remains at a near-zero value. In this case, the similarity between the original and reconstructed signals is maximized, i.e., the reconstructed signal contains all the main information characteristics of the original signal. Hence, the optimal number of modes of VMD is set at 5, which is consistent with the number of vibration events contained in the original vibration signal. Accordingly, ND method is effective for mode number selection.

## 4. Principles of SVM and OCSVM

### 4.1. SVM

**Figure 6:**
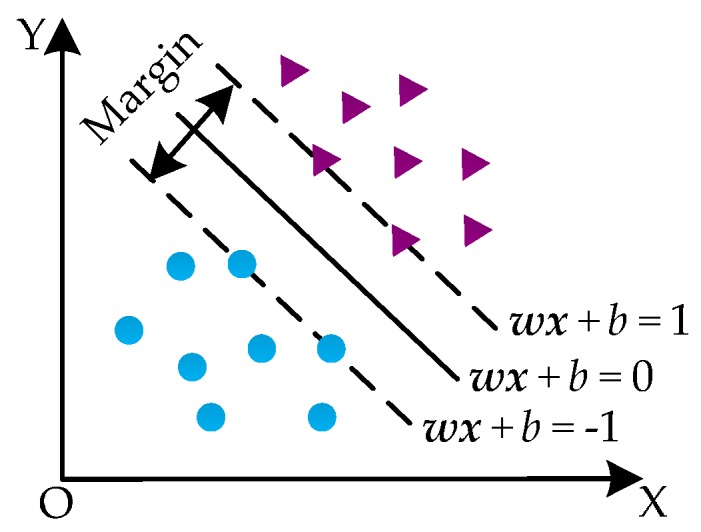
The classification principle of SVM.

SVM, proposed by Vapnik in 1995, has many advantages in solving small-sample, high-dimensional, and nonlinear pattern recognition problems [32]. The basic principles of SVM are mapping the data samples from a low-dimensional space to a high-dimensional one and making the indivisible low-dimensional data become linearly separable. A linear partition is then used to determine the classification boundary. The classification principle of SVM is shown in Figure 6.

We suppose that the training sample set $\left(x_{i},y_{i})(i=1,2,\cdots ,l;x_{i}\in R^{d},y_{i}\in \{-1,1\}\right)$ is composed of two different sample classes. The samples are linearly separable when a hyperplane w⋅x+b=0 can correctly divide them into two classes, i.e., when they satisfy:

(9) $$\left\{ \begin{array}{l} w\cdot x_{i}+b\geq 1,\;y_{i}=1\\ w\cdot x_{i}+b\leq -1,\;y_{i}=-1 \end{array}\right.,\;i=1,2,\cdots ,l,$$

The samples that satisfy $\left|w\cdot x_{i}+b\right|=1$ are called support vectors. The distance between two classes of support vectors is 2/‖w‖, i.e., the classification margin is 2/‖w‖. The goals of SVM are to seek the optimal hyperplane under the constraints in Equation (9), and make 2/‖w‖ as maximum and ${\left\|w\right\|}^{2}/2$ as minimum:

(10) $$\left\{ \begin{array}{l} \underset{w,b}{\min}\;\frac{1}{2}{\left\|w\right\|}^{2}\\ s.t.\;y_{i}\left(w\cdot x_{i}+b\right)\geq 1,\;i=1,2,\cdots ,l \end{array}\right.,$$

For most situations, the samples in the training set are linearly inseparable. SVM introduces a slack variable $\xi_{i}$ to reduce the constraint to $y_{i}(w\cdot x_{i}+b)\geq 1-\xi_{i}$. Meanwhile, penalty factor *C* is introduced to control the degree of punishment to error-classifying samples. Thus, the objective function becomes:

(11) $$\left\{ \begin{array}{l} \underset{w,b}{\min}\;\frac{1}{2}{\left\|w\right\|}^{2}+C\sum_{i=1}^{l}\xi_{i}\\ s.t.\;y_{i}\left(w\cdot x_{i}+b\right)\geq 1-\xi_{i},\;i=1,2,\cdots ,l \end{array}\right.,$$

This problem can be solved through saddle point of the Lagrange function, which is constructed as:

(12) $$L\left(w,b,\alpha_{i}\right)=\frac{1}{2}{\left\|w\right\|}^{2}-\sum_{i=1}^{l}\alpha_{i}\left[y_{i}\left(w\cdot x_{i}+b\right)-1\right],$$

where $\alpha_{i}>0$ is Lagrange coefficient. Equation (12) is converted into the following dual problem according to dual theory:

(13) $$\left\{ \begin{array}{l} \max\;Q\left(\alpha \right)=\sum_{i=1}^{l}\alpha_{i}-\frac{1}{2}\sum_{i=1}^{l}\sum_{j=1}^{l}\alpha_{i}\alpha_{j}y_{i}y_{j}\left(x_{i}\cdot x_{j}\right)\\ s.t.\;\sum_{i=1}^{l}\alpha_{i}y_{i}=0,\;0\leq \alpha_{i}\leq C \end{array}\right.,$$

The optimal solution of the quadratic programming problem $\alpha ={\left[\alpha_{1},\alpha_{2},\cdots ,\alpha_{l}\right]}^{T}$ can be obtained, followed by optimal ***w*** and *b*. The optimal decision function is:

(14) $$f\left(x\right)=\mathrm{sgn}\left[\sum_{i=1}^{l}\alpha_{i}y_{i}\left(x_{i}\cdot x\right)+b\right],$$

where sgn(*z*) is sign function, which equals +1 for z≥0 and −1 otherwise.

For a nonlinear classification problem, SVM uses kernel function ϕ(x) to map the sample data from a low-dimensional space to a high-dimensional, making these samples linearly separable. The kernel function is defined as follows:

(15) $$K\left(x_{i},x_{j}\right)=\phi \left(x_{i}\right)\cdot \phi \left(x_{j}\right),$$

After introducing the kernel function, Equation (13) becomes:

(16) $$\left\{ \begin{array}{l} \max\;Q\left(\alpha \right)=\sum_{i=1}^{l}\alpha_{i}-\frac{1}{2}\sum_{i=1}^{l}\sum_{j=1}^{l}\alpha_{i}\alpha_{j}y_{i}y_{j}K\left(x_{i},x_{j}\right)\\ s.t.\;\sum_{i=1}^{l}\alpha_{i}y_{i}=0\;,\;0\leq \alpha_{i}\leq C \end{array}\right.,$$

The decision function becomes:

(17) $$f\left(x\right)=\mathrm{sgn}\left[\sum_{i=1}^{l}\alpha_{i}y_{i}K\left(x_{i},x_{j}\right)+b\right],$$

### 4.2. OCSVM

**Figure 7:**
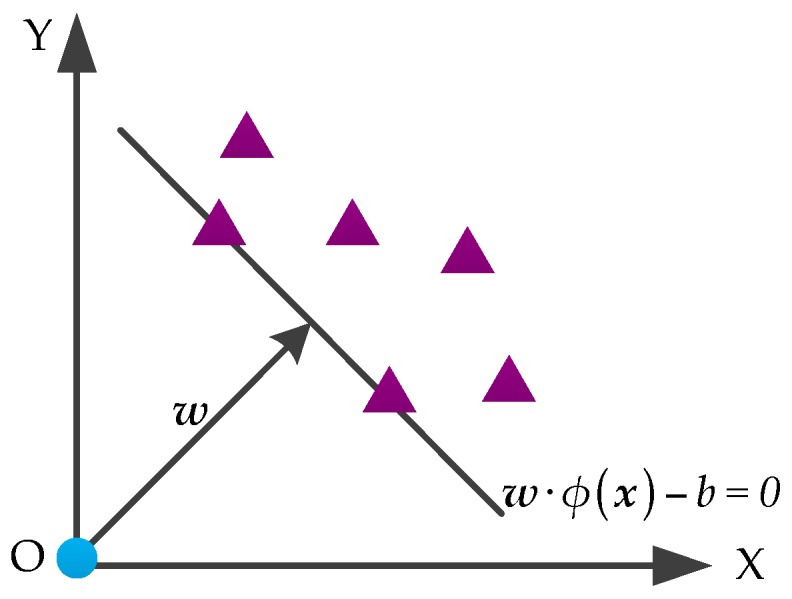
The classification principle of OCSVM.

OCSVM also maps the training data into a high-dimensional feature space by using the kernel function. OCSVM aims to separate sample data from the origin with a maximum margin, which is different from SVM. The object and no-object samples are located on either side of the hyperplane. The classification principle of OCSVM is shown in Figure 7. For convenience, we still use $\left\{x_{i}\}(i=1,2,\cdots ,l;x_{i}\in R^{d}\right)$ to represent the training sample set.

Similar to SVM, the classification hyperplane of OCSVM is expressed as w⋅ϕ(x)−b=0. OCSVM solves the following quadratic programming problem:

(18) $$\left\{ \begin{array}{l} \min\;\frac{1}{2}{\left\|w\right\|}^{2}+\frac{1}{vl}\sum_{i}\xi_{i}-b\\ s.t.\;w\cdot \phi \left(x_{i}\right)\geq b-\xi_{i},\;\xi_{i}\geq 0 \end{array}\right.,$$

where v∈(0,1] is the margin of error that controls the number of outliers. The decision function is as follows:

(19) f(x)=sgn(w⋅ϕ(x)−b),

The value of decision function *f*(***x***) is +1 or −1 along with Equation (19). *f*(***x***) is considered as the object sample when it takes the value of +1 in a test sample. Therefore, once ***w*** and *b* are solved, we can determine the sample class.

Lagrange multipliers are introduced to solve the above quadratic programming problem. The Lagrange function is as follows:

(20) $$L\left(w,\xi ,b,\alpha ,\beta \right)=\frac{1}{2}{\left\|w\right\|}^{2}+\frac{1}{vl}\sum_{i}\xi_{i}-b-\sum_{i}\alpha_{i}\left(w\cdot \phi \left(x_{i}\right)-b+\xi_{i}\right)-\sum_{i}\beta_{i}\xi_{i},$$

where $\alpha_{i},\;\beta_{i}\geq 0$ are Lagrange multipliers. We set the partial derivatives of variables w, ξ, b in Equation (20) equal to zero, yielding:

(21) $$\left\{ \begin{array}{l} w=\sum_{i}\alpha_{i}\phi (x_{i})\\ \alpha_{i}=\frac{1}{vl}-\beta_{i}\leq \frac{1}{vl},\;\sum_{i}\alpha_{i}=1\; \end{array}\right.,$$

Combined with the kernel function in Equation (15), the dual form of this optimization problem is described as:

(22) $$\left\{ \begin{array}{l} \underset{\alpha }{\min\;}\frac{1}{2}\sum_{i,j=1}^{l}\alpha_{i}\alpha_{j}K(x_{i},x_{j})\\ s.t.\sum_{i}\alpha_{i}=1,\;0\leq \alpha_{i}\leq \frac{1}{vl} \end{array}\right.,$$

The support vector is located on the hyperplane; thus *b* can be found by support vector $x_{i}$ and the corresponding $\alpha_{i}$:

(23) $$b=w\cdot \varphi (x_{i})=\sum_{j=1}^{l}\alpha_{j}K(x_{j},x_{i}),$$

The decision function together with Equation (15) can be transformed into a kernel expansion form:

(24) $$f\left(x\right)=\mathrm{sgn}\left(\sum_{i=1}^{l}\alpha_{i}K(x_{i},x_{j})-b\right),$$

Figure 6 and Figure 7 illustrate that the support vectors of OCSVM are on the classification hyperplane, whereas those of SVM are on both sides of the hyperplane with a certain distance. Accordingly, OCSVM can identify the non-target samples more accurately and has higher capability of fault identification than SVM in the fault diagnosis area of HVCBs.

## 5. Feature Extraction of Vibration Signal

### 5.1. Singular Value Decomposition (SVD)

SVD [33] is an important matrix decomposition method that is widely used in feature extraction. According to SVD theory, for an m×n matrix $A\;(A\in R^{m\times n})$, there must exist two orthogonal matrices $U_{m\times m}$ and $V_{n\times n}$, and a diagonal matrix Λ, satisfying:

(25) $$\left\{ \begin{array}{l} A=U\left[\begin{array}{ll} \Lambda & 0\\ 0 & 0 \end{array}\right]V^{T}\\ \Lambda =\mathrm{diag}\left(\lambda_{1},\lambda_{2},\cdots ,\lambda_{r}\right),\;r=\mathrm{rank}\left(A\right) \end{array}\right.,$$

where $\lambda_{i}(i=1,2,\cdots ,r)$ is the singular value of matrix ***A***, and $\lambda_{1}\geq \lambda_{2}\geq \cdots \geq \lambda_{r}\geq 0$. The singular value tends to correspond to the important information implied in the matrix, and the importance is in positive correlation with the value.

The SVD of a matrix has the following property:

We assume matrices $A,B\in R^{m\times n}$, and the singular values of ***A*** and ***B*** are $\lambda_{1}\geq \lambda_{2}\geq \cdots \geq \lambda_{R}\geq 0$ and $\sigma_{1}\geq \sigma_{2}\geq \cdots \geq \sigma_{R}\geq 0$, respectively, where R=min(m,n). Then:

(26) $$\left|\lambda_{i}-\sigma_{i}\right|\leq {\left\|A-B\right\|}_{2},\;i=1,2,\cdots ,R,$$

This property indicates that when matrix ***A*** has slight disturbance, the changes in singular values are not greater than the spectral radius of the perturbation matrix. Hence, the singular values of a matrix are insensitive to the changes in matrix elements.

### 5.2. Feature Extraction Based on LSVD

In the feature extraction of the vibration signal of circuit breakers, a few energy-based features, such as the time segmentation energy entropy (TSEE), are often used as signal features [9]. However, the energy feature of the signal is sometimes not enough to reflect the fault characteristics of the signal accurately. SVD is an effective method to extract the algebraic feature of a matrix, which can better reflect the changes in the internal characteristics of the signal.

LSVD method is used in this study to extract HVCB vibration features to improve the disturbance detection capability of SVD. A sample sequence of length *N* can be decomposed into *K* IMFs by VMD. The data length of each IMF is also *N*. Hence, the size of the IMF matrix is K×N. The research in [34] showed that the singular values of the entire matrix cannot indicate the local and detailed features of the matrix. For some faults of HVCBs, such as time delay fault, the singular values of the entire matrix tend not to reflect the fault characteristic information. Therefore, more detailed local information in the time domain is required. The local information of HVCB vibration signals at different time periods is obtained using LSVD method, which is as follows:

(1) VMD is used for decomposing HVCB vibration signals to obtain the IMF matrix.
(2) The IMF matrix is equally divided into 30 submatrices along the time axis. The size of each submatrix is K×(N/30).
(3) The 30 submatrices are decomposed by a series of SVDs, obtaining 30 singular value sequences.
(4) The singular values of each submatrix attenuate rapidly; thus the largest singular value of each submatrix *λ_i_*_max_ is selected to construct the feature vector $F=[\lambda_{1\max},\lambda_{2\max},\cdots ,\lambda_{30\max}]$.

## 6. Experimental Results

### 6.1. Feature Analysis of Measured Vibration Signal

**Figure 8:**
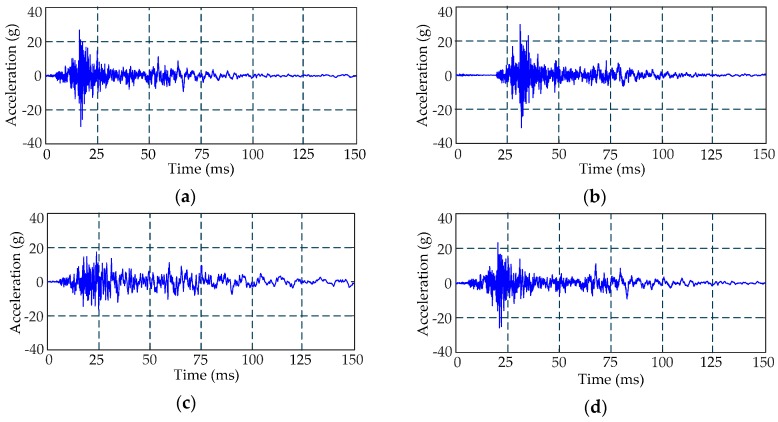
Waveforms of four types of vibration signals. (**a**) Normal condition; (**b**) Fault I; (**c**) Fault II; (**d**) Fault III.

HVCB vibration data are collected using the acquisition system in Figure 1. Three types of fault are simulated in field experiments: (1) jam fault of the iron core (Fault I); (2) looseness of the base screw (Fault II); and (3) poor lubrication of the connecting lever (Fault III). Excessive opening/closing operations will damage the circuit breaker; thus 40 samples of normal condition and 40 samples per fault type are collected through several experiments. Typical waveforms of four different types of vibration signals are shown in Figure 8.

**Figure 9:**
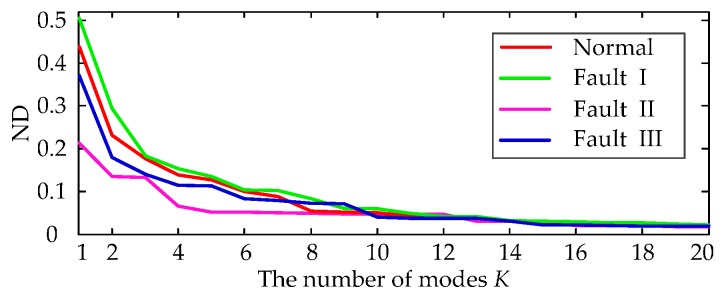
The NDs between the reconstructed signals and the measured signals with different *K*.

As mentioned previously, the number of VMD modes should be predefined. According to the abovementioned method for determining the number of modes, we use VMD to decompose the four types of vibration signals with different *K*. The NDs between their corresponding reconstructed and measured signals are then computed, which are shown in Figure 9.

Figure 9 shows that the NDs of the four signal types decrease with the increase of *K*. When *K* is greater than 10, the changes in ND values show signs of leveling off. The number of *K* modes is set to 10 to guarantee that the four signal types can be effectively decomposed.

**Figure 10:**
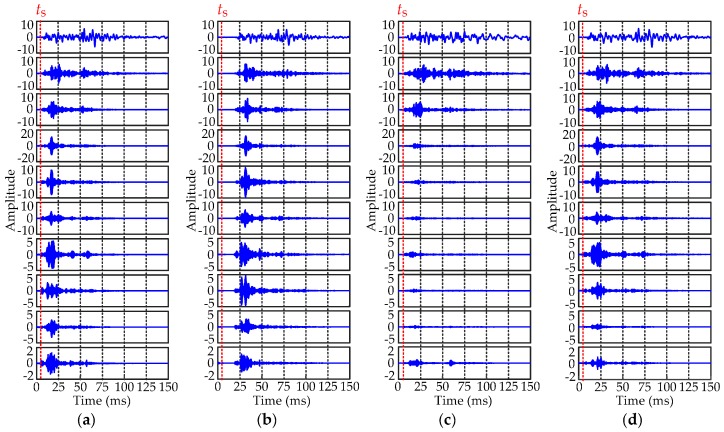
IMFs of the four types of vibration signals obtained by VMD. (**a**) Normal condition; (**b**) Fault I; (**c**) Fault II; (**d**) Fault III.

The normal and fault vibration signals are decomposed by VMD, and the corresponding IMFs are shown in Figure 10. Ten modes of each signal type are arranged from top to bottom based on the increase in center frequencies, and the red dashed line indicates the starting time *t_s_* of a normal vibration signal.

Figure 10 indicates some characteristics of fault signals in the time or frequency domain. Compared with the normal state, the vibration of Fault I has a significant time delay. The amplitudes of the last seven modes of Fault II are significantly smaller than the normal state, i.e., the vibration focuses on a lower-frequency area. The vibration time duration in different modes of Fault III is longer than other types of signals because of the poor lubrication of the connecting lever.

**Figure 11:**
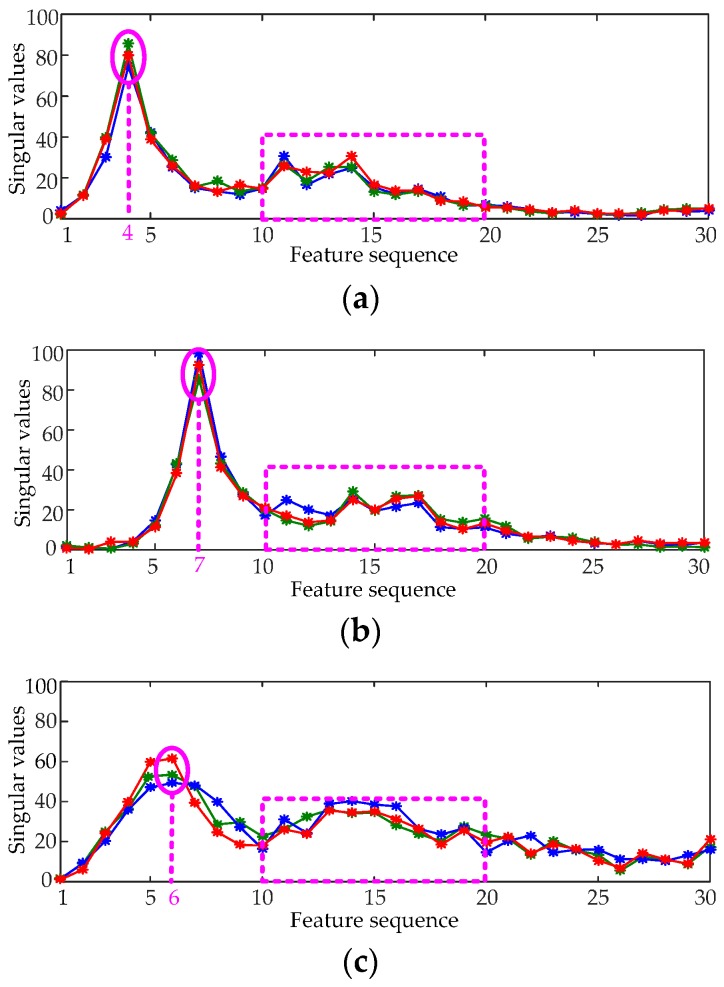

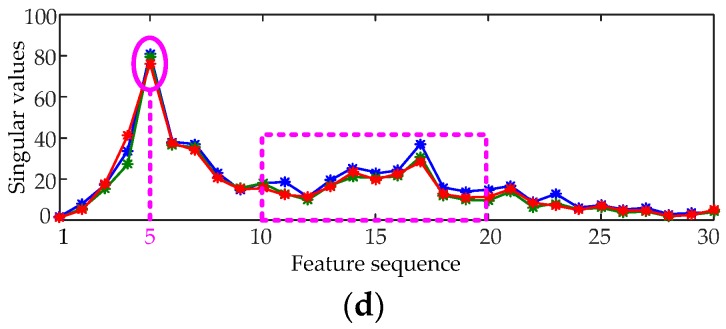
The LSV feature vectors of normal and fault signals. (**a**) Normal condition; (**b**) Fault I; (**c**) Fault II; (**d**) Fault III.

The LSVD method is adopted to extract the features of vibration signals. The LSV feature vectors of the normal and three types of fault conditions are shown in Figure 11. For clarity, each type only displays three feature vectors.

Figure 11 presents that the feature vectors of different types of vibration signals have significant differences. The peak of the feature vector of normal condition appears around the fourth feature, whereas that of Fault I appears around the seventh feature, that of Fault II appears around the sixth feature, and that of Fault III appears around the fifth feature. The variations in the 10th to 20th features of the four signals are also different. The classifier can make a good classification according to the differences among these feature vectors. These feature vectors roughly reflect the energy distributions of the corresponding vibration signals in the time domain from Figure 8 and Figure 11.

**Figure 12:**
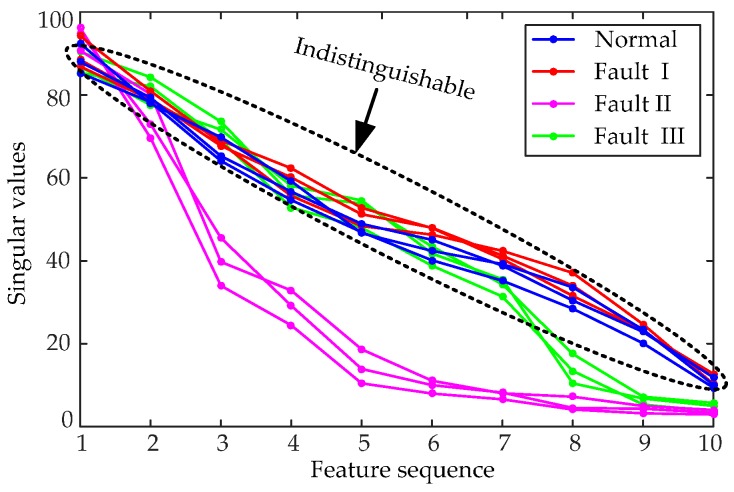
The WSV feature vectors of different types of vibration signals.

We use the whole SVD (WSVD) method to extract the features of vibration signals, validating the LSV feature vectors. The entire matrix is directly decomposed into *K* (*K* = 10 here) singular values by SVD [35]. The whole singular value (WSV) feature vectors of the four types of vibration signals are shown in Figure 12.

The WSVD method may not distinguish normal from Fault I signals, as presented in Figure 12. Fault I is essentially a time delay fault that contains the same vibration rules as normal signals. Thus, almost all the major elements of the IMF matrix of Fault I are the same as those of the normal signal. Consequently, the WSV feature of Fault I tends to be nearly equal to the normal condition. WSVD method cannot directly reflect the vibration laws of the original signal over time, unlike LSVD. Thus, LSVD approach is more suitable for the feature extraction of HVCB vibration signals.

### 6.2. Fault Classification Using MLC

The LSV feature vectors are entered into the MLC to achieve the relevant classification results. The MLC consists of three classifiers: OCSVM1, OCSVM2, and SVM. These classifiers need to be trained first. For each type of vibration signals, 40 vibration data are included. We select 20 data randomly as the training samples and the other 20 data as test samples. OCSVM1 is trained using normal training samples, whereas OCSVM2 and SVM are trained by fault training samples. SVM is the most widely used classifier in HVCB fault diagnosis and has achieved a good classification effect. We compare the classification performances of MLC and SVM. The experiment results are shown in Table 2. “New Fault” in the Table refers to the new type of fault that has not been recorded before, i.e., the unknown fault type.

According to the results in Table 2, three types of fault states are correctly recognized by the MLC method, and their classification accuracies are 100%. Conversely, three samples of Fault III are recognized as normal samples by SVM, and the corresponding classification accuracy is 85%. This comparison shows that the new approach has a higher capability of fault identification. For normal state, two samples are wrongly classified by MLC and one by SVM. For HVCBs, normal samples that are recognized as fault samples will not cause accidents and outage cost. Moreover, the operational reliability of the device is not reduced by the new method. Therefore, the new method improves the accuracy of fault diagnosis while ensuring the reliability of HVCBs. When the WSV is selected as the input feature vector of the classifier in this paper, the corresponding classification results using MLC and SVM are shown in Table 3.

The accuracy of fault diagnosis using WSVD method is lower than that using the LSVD method, as shown in Table 2 and Table 3. It illustrates that the WSVD approach is unsuitable for the feature presentation of HVCB vibration signals. Besides, the entire classification accuracy of MLC remains higher than that of SVM in such a situation.

A new fault type without training sample appearing in test samples is also considered. We assume that Fault III is the new fault, and the training samples of Fault III do not participate in the training of OCSVM2 and SVM. The classification results are shown in Table 4. The test samples of Fault III are selected as the test sample set. The classification results of MLC and SVM are compared under this situation and are shown in Table 4.

Table 4 shows that when a new fault type occurs, SVM cannot accurately identify the fault samples because of the lack of corresponding training. All fault samples are recognized as the normal state to reduce the fault diagnosis accuracy of SVM significantly. Conversely, MLC can identify a fault state with 100% accuracy. Thus, the new method has higher accuracy for the diagnosis of unknown new fault types. When a new fault is recognized, we can determine its specific fault type according to the overall report made by the maintenance personnel. In this way we can continue to accumulate fault samples and get more fault types.

## 7. Conclusions

This paper proposes a diagnosis method for HVCB mechanical faults based on VMD and MLC. The simulation and practical tests demonstrate the following advantages of the new approach:

(1) Compared with EMD, the mode decomposed by VMD has a clearer physical meaning. The latter can reduce the influence of false modes for feature extraction and has a better property of feature presentation for vibration signals.
(2) LSV can characterize the local and detailed features of vibration signals accurately, and the fault signatures can be extracted more precisely using the LSVD method, especially for delay fault.
(3) MLC uses OCSVM to improve the ability to detect fault conditions. This method can identify unknown fault types. The diagnosis accuracy and the reliability of MLC are significantly enhanced compared with those of the SVM method.

## Figures and Tables

**Table 1:** The parameter of each vibration event.

Vibration Events	*A_i_*	*t_i_* (ms)	*f_i_* (Hz)	*μ_i_*
*V*_1_	0.15	15	1200	80
*V*_2_	0.2	50	3000	50
*V*_3_	0.3	25	4500	95
*V*_4_	1.0	30	5500	75
*V*_5_	0.5	40	7000	60

**Table 2:** Diagnosis results using MLC and SVM.

Classifier	Test Sample	Diagnosis Results					Accuracy
		Normal	Fault I	Fault II	Fault III	New Fault	
MLC	Normal	18	0	0	2	0	90%
	Fault I	0	20	0	0	0	100%
	Fault II	0	0	20	0	0	100%
	Fault III	0	0	0	20	0	100%
SVM	Normal	19	0	0	1	-	95%
	Fault I	0	20	0	0	-	100%
	Fault II	0	0	20	0	-	100%
	Fault III	3	0	0	17	-	85%

**Table 3:** Diagnosis results using MLC and SVM with the WSV feature.

Classifier	Test Sample	Diagnosis Results					Accuracy
		Normal	Fault I	Fault II	Fault III	New Fault	
MLC	Normal	14	6	0	0	0	70%
	Fault I	5	15	0	0	0	75%
	Fault II	0	0	19	0	1	95%
	Fault III	0	3	0	17	0	85%
SVM	Normal	13	7	0	0	­-	65%
	Fault I	8	11	0	1	-	55%
	Fault II	0	0	20	0	-	100%
	Fault III	3	2	0	15	-	70%

**Table 4:** Diagnosis results of the new type of fault using MLC and SVM.

Classifier	Diagnosis Results				Accuracy
	Normal	Fault I	Fault II	New Fault	
MLC	0	0	0	20	100%
SVM	20	0	0	-	0

## Appendix A

As mentioned in the main text, the variational problem is written as:

(A1) $$\left\{ \begin{array}{l} \underset{\left\{m_{k}\right\},\left\{\omega_{k}\right\}}{\min}\left\{\sum_{k=1}^{K}{\left\|\partial_{t}\left[\left(\delta \left(t\right)+\frac{j}{\pi t}\right)*m_{k}\left(t\right)\right]e^{-j\omega_{k}t}\right\|}_{2}^{2}\right\}\\ s.t.\;\sum_{k=1}^{K}m_{k}=h\; \end{array}\right.,$$

The corresponding augmented Lagrange is constructed as:

(A2) $$L\left(\left\{m_{k}\right\},\left\{\omega_{k}\right\},\alpha \right)=\eta \sum_{k=1}^{K}{\left\|\partial_{t}\left[\left(\delta \left(t\right)+\frac{j}{\pi t}\right)*m_{k}\left(t\right)\right]e^{-j\omega_{k}t}\right\|}_{2}^{2}+{\left\|h\left(t\right)-\sum_{k=1}^{K}m_{k}\left(t\right)\right\|}_{2}^{2}+\langle \alpha \left(t\right),h\left(t\right)-\sum_{k=1}^{K}m_{k}\left(t\right)\rangle ,$$

The variational problem (A1) can be solved through the saddle point of the augmented Lagrange (A2). In this paper, ADMM approach is used to solve the saddle point, in which $m_{k}^{n+1}$, $\omega_{k}^{n+1}$, and $\alpha^{n+1}$ are alternately updated.

The $m_{k}^{n+1}$ is updated in the following way:

(A3) $$m_{k}^{n+1}\leftarrow \underset{m_{k}}{\arg\;\min}L\left(\left\{m_{}^{n+1}\right\},\left\{m_{i\geq k}^{n}\right\},\left\{\omega_{i}^{n}\right\},\alpha^{n}\right),$$

The value of $m_{k}^{n+1}$ can be expressed as:

(A4) $$m_{k}^{n+1}=\underset{m_{k}}{\arg\;\min}\;\left\{\eta {\left\|\partial_{t}\left[\left(\delta \left(t\right)+\frac{j}{\pi t}\right)*m_{k}\left(t\right)\right]e^{-j\omega_{k}t}\right\|}_{2}^{2}+{\left\|h\left(t\right)-\sum_{i}m_{i}\left(t\right)+\frac{\alpha \left(t\right)}{2}\right\|}_{2}^{2}\right\},$$

For simplicity, $\cdot^{n+1}$ and $\cdot^{n}$ are omitted for the fixed directions $m_{i\neq k}$ and $\omega_{k}$, respectively. They represent the most recent available updates. Problem (A4) can be solved in the frequency domain based on the Parseval-Plancherel theorem, which is as follows:

(A5) $${\hat{m}}_{k}^{n+1}=\underset{{\hat{m}}_{k},m_{k}}{\arg\;\min}\;\left\{\eta {\left\|j\omega \left[\left(1+\mathrm{sgn}\left(\omega +\omega_{k}\right)\right){\hat{m}}_{k}\left(\omega +\omega_{k}\right)\right]\right\|}_{2}^{2}+{\left\|\hat{h}\left(\omega \right)-\sum_{i}{\hat{m}}_{i}\left(\omega \right)+\frac{\hat{\alpha }\left(\omega \right)}{2}\right\|}_{2}^{2}\right\},$$

We use the variable $\omega -\omega_{k}$ instead of ω in the first term:

(A6) $${\hat{m}}_{k}^{n+1}=\underset{{\hat{m}}_{k},m_{k}}{\arg\;\min}\;\left\{\eta {\left\|j\left(\omega -\omega_{k}\right)\left[\left(1+\mathrm{sgn}\left(\omega \right)\right){\hat{m}}_{k}\left(\omega \right)\right]\right\|}_{2}^{2}+{\left\|\hat{h}\left(\omega \right)-\sum_{i}{\hat{m}}_{i}\left(\omega \right)+\frac{\hat{\alpha }\left(\omega \right)}{2}\right\|}_{2}^{2}\right\},$$

Equation (A6) can be written as the integral over the non-negative frequencies using Hermitian symmetry, which is as follows:

(A7) $${\hat{m}}_{k}^{n+1}=\underset{{\hat{m}}_{k},m_{k}}{\arg\;\min}\;\left\{\int_{0}^{\infty }4\eta {\left(\omega -\omega_{k}\right)}^{2}{\left|{\hat{m}}_{k}\left(\omega \right)\right|}^{2}+2{\left|\hat{h}\left(\omega \right)-\sum_{i}{\hat{m}}_{i}\left(\omega \right)+\frac{\hat{\alpha }\left(\omega \right)}{2}\right|}^{2}d\omega \right\},$$

The solution of the quadratic optimization problem is:

(A8) $${\hat{m}}_{k}^{n+1}\left(\omega \right)=\frac{\hat{h}\left(\omega \right)-\sum_{i\neq k}{\hat{m}}_{i}\left(\omega \right)+\frac{\hat{\alpha }\left(\omega \right)}{2}}{1+2\eta {\left(\omega -\omega_{k}\right)}^{2}},$$

Equation (A8) shows that ${\hat{m}}_{k}^{n+1}$ is the Wiener filtering of the current residual with the signal prior of $1/{\left(\omega -\omega_{k}\right)}^{2}$. ${\hat{m}}_{k}(\omega )$ can be transformed into mode $m_{k}(t)$ using inverse FT.

Similarly, $\omega_{k}^{n+1}$ is updated as follows:

(A9) $$\omega_{k}^{n+1}\leftarrow \underset{\omega_{k}}{\arg\;\min}L\left(\left\{m_{i}^{n+1}\right\},\left\{\omega_{}^{n+1}\right\},\left\{\omega_{i\geq k}^{n}\right\},\alpha^{n}\right),$$

The center frequencies $\omega_{k}$ appear only in the first term of Equation (A2). Thus, the relevant problem can be written as:

(A10) $$\omega_{k}^{n+1}=\underset{\omega_{k}}{\arg\;\min}\left\{{\left\|\partial_{t}\left[\left(\delta \left(t\right)+\frac{j}{\pi t}\right)*m_{k}\left(t\right)\right]e^{-j\omega_{k}t}\right\|}_{2}^{2}\right\},$$

This optimization problem is transformed into the Fourier domain and eventually turns into the following form:

(A11) $$\omega_{k}^{n+1}=\underset{\omega_{k}}{\arg\;\min}\left\{\int_{0}^{\infty }{\left(\omega -\omega_{k}\right)}^{2}{\left|{\hat{m}}_{k}\left(\omega \right)\right|}^{2}d\omega \right\},$$

The solution of this quadratic problem is:

(A12) $$\omega_{k}^{n+1}=\frac{\int_{0}^{\infty }\omega {\left|{\hat{m}}_{k}\left(\omega \right)\right|}^{2}d\omega }{\int_{0}^{\infty }{\left|{\hat{m}}_{k}\left(\omega \right)\right|}^{2}d\omega },$$

It shows that the new $\omega_{k}$ is the center of gravity of the power spectrum of the recent mode.

Finally, the update of $\alpha^{n+1}$ is as follows:

(A13) $$\alpha^{n+1}=\alpha^{n}+\tau \;\left(h-\sum_{k=1}^{K}m_{k}^{n+1}\right),$$

where *τ* is the update parameter of the Lagrange multiplier.
